# Hybrid heterogeneous phantoms for biomedical applications: a demonstration to dosimetry validation

**DOI:** 10.1364/BOE.514994

**Published:** 2024-01-18

**Authors:** M. Daniyal Ghauri, Stefan Šušnjar, Claudia Nunzia Guadagno, Somdatta Bhattacharya, Björn Thomasson, Johannes Swartling, Rekha Gautam, Stefan Andersson-Engels, Sanathana Konugolu Venkata Sekar

**Affiliations:** 1 Tyndall National Institute, Lee Maltings Complex, Dyke Parade, T12R5CP, Cork, Ireland; 2 Department of Engineering and Food Sciences, University College Cork, College Road, Cork, T12 K8AF, Ireland; 3 SpectraCure AB, Gasverksgatan 1, SE-222 29 Lund, Sweden; 4 Department of Physics, Lund University, P.O. Box 118, SE-221 00 Lund, Sweden; 5 BioPixS Ltd – Biophotonics Standards, IPIC, Lee Maltings Complex, Dyke Parade, T12R5CP, Cork, Ireland; 6 Department of Physics, University College Cork, College Road, Cork, T12 K8AF, Ireland

## Abstract

Phantoms simultaneously mimicking anatomical and optical properties of real tissues can play a pivotal role for improving dosimetry algorithms. The aim of the paper is to design and develop a hybrid phantom model that builds up on the strengths of solid and liquid phantoms for mimicking various anatomical structures for prostate cancer photodynamic therapy (PDT) dosimetry validation. The model comprises of a photosensitizer-embedded gelatin lesion within a liquid Intralipid prostate shape that is surrounded by a solid silicone outer shell. The hybrid phantom was well characterized for optical properties. The final assembled phantom was also evaluated for fluorescence tomographic reconstruction in conjunction with SpectraCure’s IDOSE software. The developed model can lead to advancements in dosimetric evaluations. This would improve PDT outlook as a clinical treatment modality and boost phantom based standardization of biophotonic devices globally.

## 1. Introduction

Photodynamic therapy (PDT) is a light-based therapeutic modality for cancerous as well as a number of noncancerous health conditions [1]. Dosimetry of PDT is a multi-disciplinary field relevant to planning, monitoring and evaluation of PDT clinical output [2]. It is focused on finding an optimum combination of tissue oxygenation, fluence rate, spatial distribution of photosensitizer (PS) and other factors to determine PDT outcome [3]. Depending on the targeted area, preparation for PDT involves oral, intravenous or transdermal administration of PS [1]. This administered PS is excited at an appropriate wavelength to systematically cause cytotoxicity [1]. The type and concentration of PS used depends on the specificity of the treatment area and drug resistance status of the patient [4]. An optimal PS would exhibit low dark toxicity, selectivity to target tissue, water solubility, high quantum yield in an aqueous environment and short lived photosensitivity [5]. Technological advancements in light delivery mechanisms and exploration of novel PS have increased the general acceptance of PDT as a promising modality. However, a better understanding of PDT dosimetry from cellular level to tissue response is essential for its wide clinical adaptation.

Tissue heterogeneity is inherent to many if not all treatment sites in the body. These anatomical features create challenges for accurate PS dosage estimations and fiber placements for light delivery. This can lead to adverse outcomes such as retention in non-target tissues or tumor relapse due to incomplete light delivery or insufficient accumulation of PS at treatment site [6]. This limits the clinical effectiveness of PDT [4]. Tissue phantoms that consider tissue heterogeneity will be helpful in providing realistic scenarios of PS distribution and thereby indirectly help in understanding the PS diffusion once applied in real tissue. Phantoms are already widely used in developing biomedical technological solutions [7,8]. In the case of PDT along with the understanding of spatial distribution, incorporating PS into these phantoms could potentially provide insights into precision, predictability and reliability of real-time dosimetric evaluations [9,10]. 3D printing technologies have been reported in the literature to create anthropomorphic phantoms of various anatomical sites [11–14]. However, despite realistic representation, they fail to incorporate diverse well calibrated optical properties [15].

Anthropomorphic optical phantoms having specific optical properties have been documented in the literature [15,16]. However, replicating optical properties and anthropomorphic features into a phantom for PDT application despite technological advancements still presents various degrees of challenges. Polymer-based solid phantoms with specific optical properties lack the flexibility to change PS concentration and fiber positions [17]. Moreover, inserting additional fibers or movement of already present fibers during the course of study may introduce air pockets which can lead to erroneous results. Additionally, widely used PS are water soluble and not soluble in curing materials like silicone or epoxy [18]. On the other hand, liquid phantoms address these limitations but lead to difficulties in creating tissue heterogeneities and also suffer from a relatively short shelf life [19]. For interstitial PDT applications using liquid phantoms necessitate additional support structures for mounting fibers at specific positions. A phantom model that could address the above-mentioned challenges can enable optimization and bring innovation in PDT dosimetry.

In this study, we present a hybrid heterogeneous prostate PDT phantom combining the strengths of both solid and liquid phantoms. A gelatin-based solid tumorous region inside a liquid Intralipid “prostate” surrounded by a solid silicone outer shell was designed and fabricated. The water-based gelatin tumor phantom allows utilization of widely used PS such as Visudyne and offers the possibility of changing PS concentration. The use of an Intralipid liquid prostate avoids the introduction of air bubbles at the interface of tumor and surrounding phantom. A solid silicone outer shell enables the mounting of fibers at specific locations. The ease of manufacturing, tunability of tumor geometry and PS concentration enhance its utility for realistic heterogeneous tissue phantom studies for PDT dosimetry. The trend of PS concentration in phantoms was evaluated using fluorescence capabilities. The optical properties of the phantom recipe were characterized using time-domain diffuse optical spectroscopy. A preliminary evaluation of the tomographic reconstruction of the hybrid phantom is demonstrated.

## 2. Material and methods

### 2.1 Structure of hybrid phantom

**Fig. 1.**
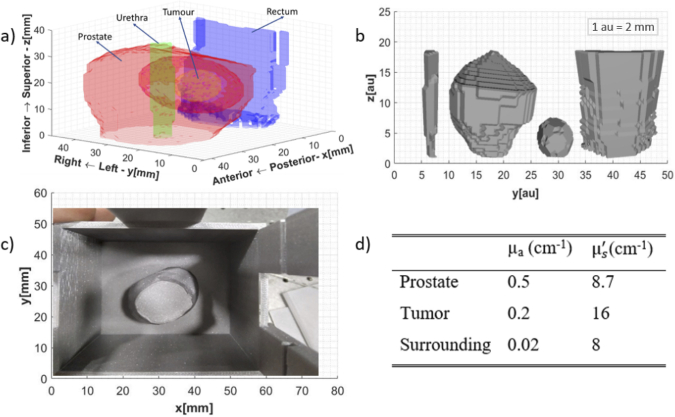
(a) Tissue 3D structural layout based on the ultrasound data (b) CAD files for the anatomical features identified (Left to Right: Urethra, Prostate, Tumor, Rectum,) (c) Outer mold for the silicone phantom showing the placement of prostate inside(d) Optical properties at 690 *nm* [21,22].

Prostrate tissue phantoms were created based on real patient ultrasound data [20]. Segmentation of various tissue structures from the data is presented in Fig. 1(a). Figure 1(b) illustrates their respective computer-aided design (CAD) files.

To reduce the fabrication challenges urethra and rectum were no longer considered the tissues of interest. The final hybrid phantom realized has three distinctive regions: tumor, prostate, and surrounding tissue. The gelatin-based tumor is inside an Intralipid-filled prostate enclosed by solid silicone surrounding tissue. This assembling of the three distinctive phantoms is in accordance with the geometry identified in Fig. 1(a). The respective molds of these three structures for phantom manufacturing were printed using a standard 3D printer (Original Prusa i3 MK3S+, PRUSA Research). The optical properties of the three distinctive regions were taken from the literature [21,22] and are tabulated in Fig. 1 (d).

### 2.2 Phantom recipe & fabrication

**Chemicals:** Gelatin (G2500, Sigma-Aldrich USA), Intralipid 20% w/v (Fresenius Kabi, Ltd., Germany), India ink (Higgins, 44201 Chartpak Inc, USA), Carbon Black [18] (QL3261, Polycraft Black Silicone Pigment, UK), Titanium dioxide [18] (248576,Sigma-Aldrich, USA), Visudyne (Cheplapharm Arzneimittel GmbH, Germany). All solutions were prepared using deionized water.

**Fabrication – Solid Silicone Phantom:** The solid silicone outer shell was fabricated by following the process documented by Konugolu Venkata Sekar *et al* [18]. Briefly, silicone was polymerized using carbon black and titanium oxide as absorber and scatterer respectively. The steps including stirring, ultrasound, vacuum and others were closely followed [18]. Figure 1 (c) shows the final mold and placement of the prostate for realizing the desired silicone phantom. In addition, a bulk phantom (cylinder – diameter 8.3 *cm* and thickness 4 *cm*) was fabricated from the same mixture for time domain optical properties characterization.

**Fabrication - Liquid Intralipid Phantom:** Liquid Intralipid prostate tissue phantom was realized by first diluting India ink in water to achieve 0.76% dilution. This served as the stock for realizing liquid phantom. Subsequently, 2.0 *g* from this stock and 4.4 *g* Intralipid were mixed with water to achieve a final volume of 200 *g* for attaining optical properties of prostate stated in Fig. 1 (d).

**Fig. 2.**
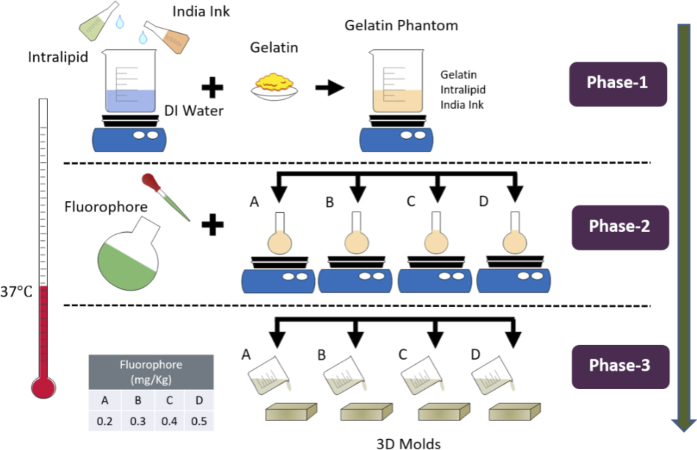
Synthesis process of gelatin phantoms.

**Fabrication – Gelatin Phantom:** Gelatin tumors were prepared by modifying the development procedure in literature to suit our application [23]. A temperature of 37 °C was monitored using a digital thermometer and maintained continuously throughout the phantom making process. The synthesis process followed to achieve optical properties (as mentioned in Fig. 1 (d)) is illustrated in Fig. 2. Initially, 0.49% India ink stock solution was prepared in water. Then 287.4 *g* of water was transferred in an adequate glass beaker and placed on a hot plate to raise temperature to 37 °C and continuously stirred at 300 rpm. Once the desired temperature was achieved 5 more minutes were allowed to achieve homogenous temperature conditions. Afterwards, 51 *g* of Intralipid and 3.3 *g* of India ink from the stock solution were added. After ∼5 minutes 39.6 *g* of gelatin powder was slowly added to the beaker to avoid cluster formation. The mixture was continuously stirred for 20 more minutes to ensure a uniform blend. This point marks the completion of Phase 1 of making gelatin phantoms.

In Phase 2, a Visudyne solution was initially prepared to achieve 0.01 *mg/ml* concentration of verteporfin (an active substance in Visudyne) as fluorophore stock solution. From the final blended solution realized in Phase 1, 28 *g* was transferred into each of four separate 50 *ml* glass beakers (A, B, C, D) kept on a hot plate under constant stirring. Fluorophore from stock was added in the amounts of 0.60, 0.90, 1.20 and 1.50 *g* to beaker A, B, C, D respectively to achieve dosage of 0.20, 0.30, 0.40, 0.50 *mg/kg* (fluorophore concentration in *mg* / phantom mass in *kg*). Deionized water was added in each beaker to make up the final mass of 30 *g*. The remaining solution in the main beaker was used for making two phantoms as control groups. The first control group phantom was created by transferring 28 *g* of the blended solution in Phase 1 to another beaker and adding 2.0 *g* water. Whereas for the second control group 230 *g* of the blended solution and 16 *g* of water was added to create bulk phantom for optical properties characterization. For each of these solutions, series A to D and the control group phantoms, 15 minutes were allowed to ensure adequate mixing. Afterwards, the phantoms were sonicated for 1 minute to remove air bubbles.

In Phase 3, the realized phantoms were poured into the respective molds. After ∼5 minutes these molds were placed in the freezer at -20 °C for 1 hour to solidify. The phantoms were then stored in the refrigerator at 4 °C in the air and light tight mold until further analysis and use.

### 2.3 Instrumentation

After the curing process, the optical properties of the three realized bulk phantoms were characterized at 690 *nm* using a time domain diffuse optical spectroscopy system [24,25]. The system consists of pulsed supercontiuum laser (20 MHz rep. rate, 400 to 1750*nm*, SC450, Fianium, United Kingdom), the wavelength selection was achieved using Pellin Broca prism and the selected wavelength is coupled into a 50 *μm* multimode source fiber. The detection system consists of a 200 *μm* multimode detection fiber coupled to a SPAD (SPAD, PDM-100 *μm* active area, MPD, Italy) detector. The photon counts from detector were histogrammed using time to digital converter (TDC, Picoharp 300, Picoquant, Germany) to generate photon time of flight (pTOF) curves. The measurements were performed in reflectance geometry at 2 *cm* source detector distance.

**Fig. 3.**
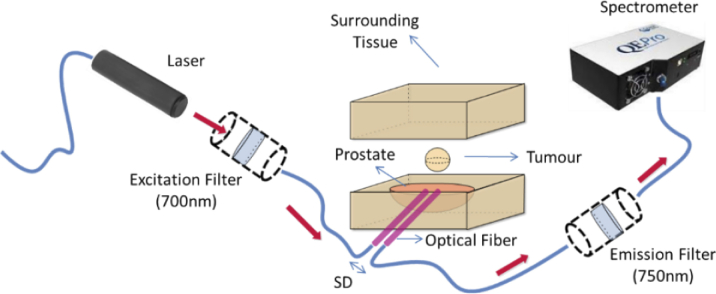
Schematic of optical arrangement used for characterizing gelatin phantoms.

Next, the three distinctive regions were assembled together such that the gelatin-based tumor is inside an Intralipid filled prostate enclosed by solid silicone surrounding tissue similar to as shown in Fig. 3. This phantom geometry is in line with the various tissue structures already presented in Fig. 1(a). Then, the realized gelatin phantoms representing tumors were subjected to interstitial optical measurements to characterize the diffuse fiber to fiber fluorescence signal as illustrated in Fig. 3. Optical fibers, mimicking transperineal insertion, were placed into the hybrid model. A 5.0 mm source-detector (SD) distance was maintained between the two fibers. Figure 3 illustrates the configuration of the optical system for characterizing the fluorescence capabilities of the phantom. It comprises an illumination source, associated filters and a spectrometer. Illumination was achieved by a 690 *nm* laser diode, a short-pass 700 *nm* excitation filter (FESH0700, Thorlabs), a long-pass 750 *nm* emission filter (FELH0750, Thorlabs), at 750 *nm* and a UV-Vis spectrometer (QEPro, Ocean Optics).

### 2.4 Tomographic reconstruction for PDT

**Fig. 4.**
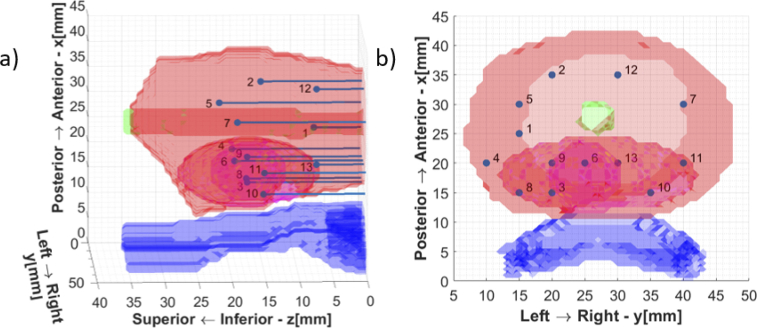
Fiber positions for the tumor geometry (a) Lateral View (b) Top View.

SpectraCure’s P18 system for interstitial PDT along with IDOSE software was used for determining fiber placement positions and data collection as well [26,27]. For light delivery and monitoring for this particular prostate and tumor geometry the algorithm identified a minimum of 13 bare-end optical fibers for treatment (point source illumination and light collection). Hence, the final assembled phantom was subjected to interstitial light delivery using 13 fibers. The fiber positions identified are illustrated in Fig. 4.

Needle piercings were made at these locations in the silicone phantom for inserting optical fibers inside. The urethra and the rectum are just shown for orientation purposes, as already stated they are not present in the final realized phantom.

The diffuse fluorescence tomographic reconstruction methods were developed in Matlab using NIRFAST package [28,29] and previous work by Axelsson *et al* [30]. In the current implementation, the space is represented by a finite element mesh with around 26,000 nodes. The distribution of nodes was such that more nodes were placed in regions around the fibers, along all three axes, compared to the periphery of the medium. It is required to solve for ∼26,000 unknowns (fluorescent absorption coefficients) with only 156 data points (obtained by 13 fibers acting as both source and detector – all possible source-detector pairs). Every data point is a ratio between the fluorescent signal compared to the excitation signal, known as normalized Born ratio [31]. The excitation and fluorescent signals are collected by the same fiber, when one of the other 12 fibers is emitting light.

A vector of measurements M contains all 156 data points and is compared to the forward model F(η) for given values of fluorophore yields η in all mesh nodes. Fluorophore yield in each node is a product of the fluorophore absorption coefficient in that node $\mu_{af}$ , and fluorescence quantum yield γ : $\eta =\mu_{af}\gamma$ . Forward model is calculated from the diffusion equation, as described in Ref. [30]

(1) $$F_{s,d}(\eta )=\;\frac{1}{U_{x}(\vec{r_{s}},\vec{r_{d}})}\sum_{n=1}^{N_{n}}U_{m}(\vec{r_{d}},\vec{r_{n}})U_{x}(\vec{r_{s}},\vec{r_{n}})\Delta V\eta_{n}$$

where $U_{x,m}(\vec{r_{s}},\vec{r_{d}})$ is the forward solution for excitation light (*x*) or fluorescent emission light (*m*), at the position of the source $\vec{r_{s}}$ , detector $\vec{r_{d}}$ or node $\vec{r_{n}}$ , ΔV is the element volume, $\eta_{n}$ is the fluorophore yield in node *n*, and the number of nodes is $N_{n}.$

The inverse problem consists of finding the vector of fluorophore yields, or fluorophore absorption coefficients in all nodes such that the difference between the measurements M and the forward model F(η) is minimized. Since the problem is ill-posed, Tikhonov regularization is applied, such that the objective function to minimize becomes [30]

(2) $$\Omega =||M-F\left(\eta \right)|{|}^{2}+\lambda ||L\left(\eta -\eta_{0}\right)|{|}^{2}$$

In this implementation, regularization matrix L was set to identity matrix, i.e., no geometrical prioring was done, since the emphasis was to demonstrate the usefulness of the fluorescent signal obtained from hybrid phantoms, not to geometrically bias the solution to have a more accurate quantitative reconstruction. Initial guess for the fluorophore yields in all nodes was set to 0, therefore, $\eta_{0}=0$ **.** A variant of Levenberg-Marquardt algorithm was applied for numerical convergence to a solution in iterative steps. Regularization parameter λ is set to the maximum of the diagonal of the Hessian matrix $H=J^{\text{T}}J$ , with $J=\frac{\partial F(\eta )}{\partial \eta }\;$ being Jacobian matrix, calculated at the beginning of each iteration. In each iteration, λ is scaled according to new Hessian matrix values, and additionally divided by a factor $\sqrt[{4}]{10}$ . After each iteration *i*, fluorophore yields vector takes the updated value [30]

(3) $$\eta_{i}=\eta_{i-1}+{\left(J^{\text{T}}J+\lambda I\right)}^{-1}J^{\text{T}}(M-F(\eta_{i-1}))$$

The iterative method stops when the decrease of the square of the 2-norm of the error $\|M-F{\|}^{2}$ is less than 2%, or maximum number of 20 iterations is reached.

More general reconstruction methods, compared and analyzed in simulations, and finally validated on a series of standardized phantoms described in this work, will be a subject of future publications.

## 3. Results & discussion

This study presents the design and development of a hybrid heterogeneous prostate phantom combining the strength of both the solid as well as liquid phantom for PDT application.

### 3.1 Phantom characterization

The three distinctive bulk phantoms were characterized in collaboration with BioPixS using the state-of-the-art time domain diffuse optical spectroscopy system [24]. The characterization results are tabulated in Table 1 along with the uncertainty of optical properties (absorption, reduced scattering) measurements. The properties can be followed up using the trace code: BioPixS0014.

**Table 1.** Optical properties of the final realized phantoms

Wavelength	Tissue Type	Absorption	Scattering	Absorption Coefficient of Variation	Scattering Coefficient of Variation
nm		cm^-1^	cm^-1^	%	%
690	Surrounding	0.03	9.1	2	1
720	Surrounding	0.03	8.6	1	1
780	Surrounding	0.03	8.2	1	1

690	Tumor	0.20	15.7	0.2	1.2
720	Tumor	0.20	15.0	0.0	1.0
780	Tumor	0.20	13.7	3.1	3.0

690	Prostate	0.51	7.3	1.0	0.7
720	Prostate	0.51	7.1	0.6	0.4
780	Prostate	0.48	6.7	3.1	3.1

**Fig. 5.**
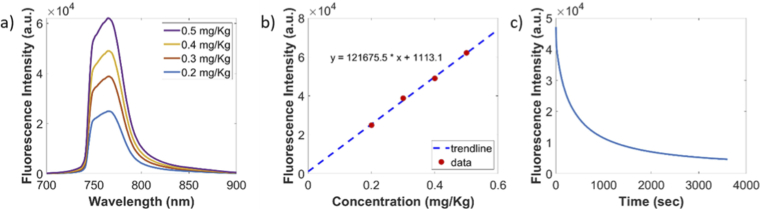
Background Corrected: (a) Fluorescence emission results (b) Proportionality plot between fluorescence intensity and PS concentration (c) Photobleaching effect in gelatin phantom.

The diffuse fluorescence signal from the phantom was studied at 690 *nm*. The fluorescence response of the phantom to the changing concentrations of the photosensitizer after background correction is depicted in Fig. 5 (a) and (b). The coefficient of variation (CV) for three repetitions was found to be less than 3% which is the expected CV of the system [18]. The gelatin tumor with 0.4 *mg/kg* concentration of verteporfin was subjected to PDT-like light delivery at 80 *mW* for one hour. The photobleaching effect was observed as depicted in Fig. 5 (c).

Wavelength selection for PDT is limited to the therapeutic window however wavelengths higher than 800 *nm* are generally insufficient to create the photodynamic effect on its own [3]. Most clinical PS are therefore illuminated within 600 *nm* to 800 *nm* range [3]. The selection of 690 *nm* as excitation source was made to match the excitation wavelength for verteporfin. For a photosensitizer with a different excitation wavelength, the same hybrid approach can be followed for phantom manufacturing with changes in concentrations of scatterer and absorber respectively to match the optical properties at that particular wavelength. Research effort targeted at exploring innovative materials and recipes has been reported in the literature for the development of multi-wavelength tissue phantoms [32]. However, despite their appeal the manufacturing process of such phantoms is not easy. In the interest of a simple fabrication process the proposed phantom has been limited to one wavelength only i.e., 690 *nm*.

### 3.2 Tomographic reconstruction: preliminary results

**Fig. 6.**
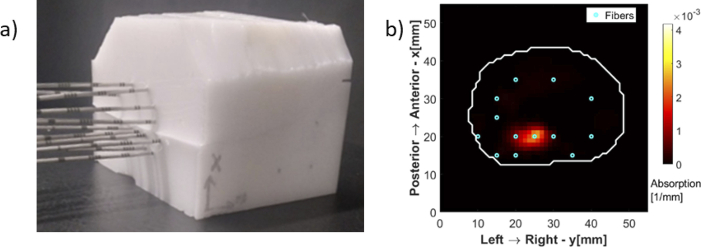
(a) Final assembled hybrid phantom ready for measurement (b) Tomographic Reconstruction.

The final assembled phantom was subjected to PDT-like light delivery using 13 fibers as shown in Fig. 6 (a). The experimental studies performed resemble a simplified but anatomically realistic model of light propagation inside the human prostate. The preliminary reconstruction result is shown in Fig. 6 (b) for one selected slice corresponding to the center of the fluorophore sphere ( z=18mm ). A homogeneous quantum efficiency of 10% for fluorescence from verteporfin was assumed to reconstruct the fluorophore absorption coefficient [33,34]. The white contour shows the interface between the prostate and the surrounding tissue, or in the case of the phantom, between the liquid part and the solid silicone outer shell. The reconstructed fluorophore spatial distribution in the chosen plane matches the corresponding cross section of the gelatin tumor sphere in the hybrid phantom.

### 3.3 Manufactured phantom & validation

The hybrid phantom constitutes a gelatin-based solid tumorous region inside a liquid prostate surrounded by a solid silicone outer shell. The phantom recipe for each of the distinctive organs assumes homogenous optical properties throughout. The photosensitizer selected was verteporfin because it is currently being investigated for use in prostate cancer PDT [35,36].

**Fig. 7.**
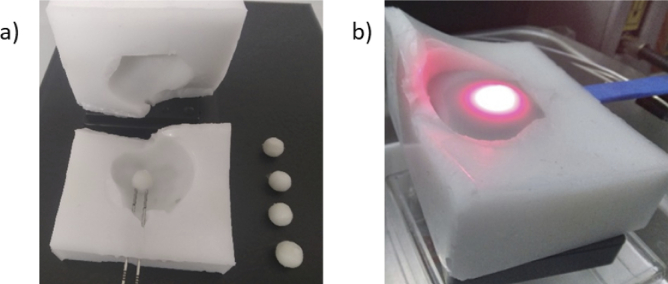
(a) Tumors for varying concentrations of verteporfin along with outer shell (b) Light delivery in gelatin tumor surrounded by liquid prostate within the lower half of the silicone outer shell.

Phantom development was an iterative process with each iteration further refining the recipe as well as minimizing occurrences of human error. The gelatin tumor phantoms with different concentrations of verteporfin along with the control group and the surrounding silicone phantom are depicted in Fig. 7 (a).

The final assembly of the phantom involves tumor placement inside the prostate region and the insertion of optical fibers. While inserting optical fibers mimicking transperineal insertion inside the gelatin tumor it might change orientation or get deformed due to external pressure. A similar effect is also exhibited by the prostate during actual PDT sessions [37]. The surgeons rely on ultrasound or magnetic resonance imaging (MRI) for transperineal insertion of optical fibers to ensure that fibers are placed correctly and at the same time minimize damage to the surrounding organs such as urethra, rectum etc. [37]. In our case use of liquid phantom as prostate overcomes the deformation and displacement issue while inserting fibers. On the other hand, the gelatin phantom does suffer from this issue. However, since the outer silicone shell is divided into two halves the operator can simply remove the upper half of the outer silicone shell, insert the fiber and then place the gelatin phantom in the fiber at a specific location. No slippage of gelatin tumors from the fiber was observed even without liquid prostate. Once satisfied by the position of the gelatin tumor the operator can fill it with liquid prostate as shown in Fig. 7 (b). Then, the upper half of the silicone outer shell needs to be placed back. Afterwards, the rest of the liquid phantom was injected into the prostate cavity using a syringe through one of the needle piercings in the outer silicone shell. Slight leakages were observed from the phantom when the syringe was taken out and fiber was placed in that needle piercing as well. During and after the course of measurements no change in position of gelatin phantom was observed.

The solid silicone outer shell keeps the inner phantoms and the position of fibers at the right location without the need of any additional support. The benefit of having a liquid phantom inside the solid silicone outer shell is that the translational or rotational movement of fibers during the course of the study does not introduce air gaps. In comparison to a fully solid heterogeneous phantom, this hybrid approach offers a way to ensure that the optical properties do not get modified during the study leading to erroneous results. The use of liquid prostate also offers the possibility to accommodate changes in tumor geometry as well as the possibility to tailor tumor placement anywhere in the prostate. The selection of gelatin for mimicking the tumorous part was done because it is a widely available gelling material. Gelatin phantoms are biocompatible, resemble human tissues closely and allow the incorporation of water soluble photosensitizer [38]. Gelatin also offers a simpler manufacturing process that can be pursued even in a low resource setting. If needed a change in tumor geometry can be achieved by using a different custom-made mold during the phantom making process. Although verteporfin was used as the photosensitizer for the demonstrated phantom, the same development and quantification process can be followed for testing the efficiency of other photosensitizers. Both of these scenarios allow for reusing other parts of the phantom. All these factors add to the attractiveness of using gelatin to mimic a tumor for PDT application.

### 3.4 Limitations

During PDT-like light delivery measurements using 13 fibers, instances of leakages of the liquid phantom were observed from the region where the distance between the prostate boundary and the outer boundary of the silicone outers shell was small. A container similar to Fig. 1 (c) was printed using the same 3D printer to submerge the final assembled phantom in the liquid phantom to ensure that no instances of leakages interfere with measurements. The phantom design of the silicone outer shell will be further evolved to resolve this issue.

The ideal shelf life of gelatin phantoms was observed to be almost twenty days under optimum storage conditions after which they start to dry out or start microbial growth. Almost the same shelf life has been reported by Gautam *et al* [23]. Improved storage mechanisms or the addition of preservatives could potentially further enhance shelf life. In the case of the latter, care must be taken to ensure that the optical properties of the phantom are not affected. Also, following up on the hybrid phantom design further studies can be done with the incorporation of more anatomic sites such as urethra and rectum. Although the CAD files were generated for these structures as well, these were omitted from this proof-of-concept study to keep complexity to a minimum. The approach to incorporate urethra and rectum will be to make both urethra and rectum as solid phantoms. The prostate surrounding phantom will be modified to provide space for the urethra and rectum solid structures which will be assembled together, and prostate liquid phantom will be filled in the end. Additionally, the outer silicone shell can be reshaped to reproduce the entire pelvic geometry. The mechanical properties of the outer phantom can be tuned to be similar to the perineum so that the same resistance is felt when inserting optical fibers for prostate PDT. Such a phantom has the potential to serve as a training platform for clinicians/surgeons for practicing or pedagogical purposes. This would also assist in improving robotic interventions.

## 4. Conclusions

The design, manufacturing and characterization of a hybrid solid-liquid prostate tissue phantom has been reported in this study. The hybrid approach combines the benefits of both types of phantoms to present an anatomically realistic patient specific treatment site model. The proposed phantom contains a gelatin tumor inside a liquid prostate enclosed in a silicone outer shell representing surrounding tissue. Use of gelatin for making tumors offers adjustable photosensitizer concentration, customizable tumor geometry and easy needle placement for PDT application. Liquid prostate ensures that any fiber movements during measurements do not lead to changes in optical properties due to air gaps. While the silicone based surrounding tissue helps the mounting of the fibers at specific locations. Gelatin tumor characterization demonstrated a directly proportional trend between fluorescence signal and PS concentrations as well as photobleaching effect. Preliminary fluorescence tomographic reconstruction results using SpectraCure IDOSE software are promising. Although the hybrid phantom has been demonstrated herein for prostate cancer, it has potential to be applied for evaluating other treatment sites in the body as well. Reliable dosimetry assessment is central to positive treatment outcome. It is anticipated that the use of the proposed heterogeneous phantom would facilitate further development and optimization of reconstruction algorithms for improved dosimetry evaluations.

## Data Availability

Data underlying the results presented in this paper are not publicly available at this time but may be obtained from the authors upon reasonable request.
